# Exploring the association between Morgellons disease and Lyme disease: identification of *Borrelia burgdorferi* in Morgellons disease patients

**DOI:** 10.1186/s12895-015-0023-0

**Published:** 2015-02-12

**Authors:** Marianne J Middelveen, Cheryl Bandoski, Jennie Burke, Eva Sapi, Katherine R Filush, Yean Wang, Agustin Franco, Peter J Mayne, Raphael B Stricker

**Affiliations:** International Lyme and Associated Diseases Society, Bethesda, MD USA; Department of Biology and Environmental Science, University of New Haven, West Haven, CT USA; Australian Biologics, Sydney, NSW Australia; 450 Sutter Street, Suite 1504, San Francisco, CA 94108 USA

**Keywords:** Morgellons disease, Lyme disease, *Borrelia burgdorferi*, Spirochetes, Dermopathy

## Abstract

**Background:**

Morgellons disease (MD) is a complex skin disorder characterized by ulcerating lesions that have protruding or embedded filaments. Many clinicians refer to this condition as delusional parasitosis or delusional infestation and consider the filaments to be introduced textile fibers. In contrast, recent studies indicate that MD is a true somatic illness associated with tickborne infection, that the filaments are keratin and collagen in composition and that they result from proliferation and activation of keratinocytes and fibroblasts in the skin. Previously, spirochetes have been detected in the dermatological specimens from four MD patients, thus providing evidence of an infectious process.

**Methods & Results:**

Based on culture, histology, immunohistochemistry, electron microscopy and molecular testing, we present corroborating evidence of spirochetal infection in a larger group of 25 MD patients. Irrespective of Lyme serological reactivity, all patients in our study group demonstrated histological evidence of epithelial spirochetal infection. Strength of evidence based on other testing varied among patients. Spirochetes identified as *Borrelia* strains by polymerase chain reaction (PCR) and/or in-situ DNA hybridization were detected in 24/25 of our study patients. Skin cultures containing *Borrelia* spirochetes were obtained from four patients, thus demonstrating that the organisms present in dermatological specimens were viable. Spirochetes identified by PCR as *Borrelia burgdorferi* were cultured from blood in seven patients and from vaginal secretions in three patients, demonstrating systemic infection. Based on these observations, a clinical classification system for MD is proposed.

**Conclusions:**

Our study using multiple detection methods confirms that MD is a true somatic illness associated with *Borrelia* spirochetes that cause Lyme disease. Further studies are needed to determine the optimal treatment for this spirochete-associated dermopathy.

## Background

**Figure 1:**
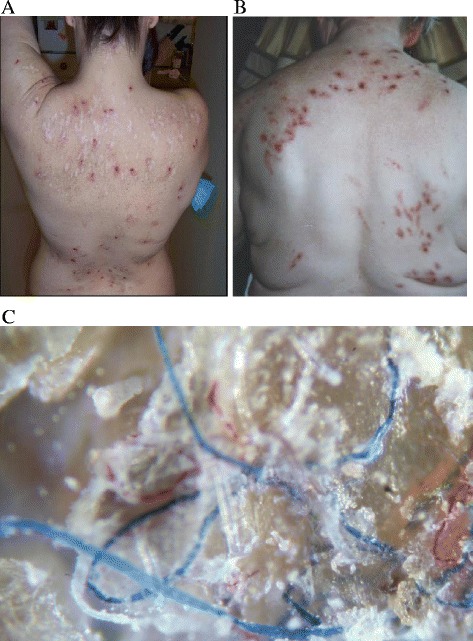
**Clinical features of Morgellons disease. A**, MD patient back showing lesions covering entire surface, including areas out of patient’s reach. **B**, Back of patient with scratching-induced lesions showing distribution limited to patient’s reach. **C**, Multicolored fibers embedded in skin callus from MD Patient 2 (100x). **B** reproduced from Reference 19, used with permission of the publisher.

Morgellons disease (MD) is a complex dermopathy characterized by the spontaneous appearance of slowly-healing skin lesions that contain multicolored filaments either lying under, embedded in, or projecting from skin (Figure 1A-C) [1-9]. Patients may also exhibit constitutional, musculoskeletal and neurocognitive symptoms that are associated with Lyme disease (LD) and tickborne coinfections. The presence of these symptoms suggests an infectious etiology of the dermopathy and possible vectoring by ticks [4,7,8].

Previous studies found that MD patients demonstrate seroreactivity to *Borrelia burgdorferi* (Bb) antigens and multisystemic symptoms consistent with LD, suggesting a spirochetal etiology [4,7,8]. In addition, histological, electron microscopic and PCR studies of dermatological tissue containing filamentous inclusions from four MD patients confirmed the presence of *Bb sensu stricto* spirochetes [6,7]. Successful bacterial culture of motile spirochetes in BSK-H medium inoculated with MD dermatological tissue demonstrated the viability of spirochetes in two of these patients, and one culture was confirmed as *Bb sensu stricto* by PCR analysis [7]. A case study of an MD patient in Australia reported that endpoint PCR and Basic Local Alignment Search Tool (BLAST) analysis resulted in the detection and identification of *Borrelia garinii* [8]. These preliminary studies suggest that MD may be a particular manifestation of LD and that strains of *Bb sensu stricto* and *Bb sensu lato* are implicated as etiologic agents [7,8].

In light of the preliminary studies indicating an association of Lyme spirochetes with MD, we undertook a histological, electron microscopic and DNA study of North American MD patients to investigate the presence of borrelial spirochetes systemically and in dermatological specimens. Culture was also undertaken to establish if spirochetes detected in MD tissue were viable organisms and to determine whether *Borrelia* infection in these patients is systemic.

## Methods

### Patient selection

All patients included in this study met the key diagnostic criterion documented by a healthcare provider: the presence of fibers that were visible underneath unbroken skin or that were embedded in or projecting from skin. Patients were selected from across Canada and the USA, and they were included in the chronological order in which they volunteered. No patients were excluded from study participation provided that they had sample material that was suitable for study and provided that they met the diagnostic criterion. Written informed consent for participation in the study was obtained from all participants. Approval for sample collection was obtained from the Western Institutional Review Board, Olympia, WA. Further approval for sample testing was obtained from the Institutional Review Board of the University of New Haven, West Haven, CT.

A diagnosis of Lyme disease or positive Lyme serologic testing prior to study participation was not a requirement. Some patients had a prior Lyme diagnosis or serologic testing for Lyme disease while others did not, as shown in Table 1. Those who were not tested with Lyme serology prior to the study were encouraged to be tested, but did so after samples for the study had been collected. Some patients declined to have Lyme serologic testing performed. Some of the subjects had received antibiotic therapy for Lyme disease but were not receiving treatment during the time of sample collection. Two subjects, 1 and 12, were currently taking antibiotics during the time of sample collection.

**Table 1:** **Clinical data for study patients**

**Patient #**	**Age**	**Gender**	**Lyme serology**	**Coinfecting tickborne diseases**	**Antibiotic treatment**
1	86	Female	Positive	*Babesia microti, Bartonella henselae*	Treated at time of sampling
2	52	Female	Positive	*Ehrlichia chaffeensis,* *Anaplasma phagocytophilum*	Previous treatment, but off at time of study
3	56	Female	*Positive	Not tested	Never treated
4	75	Female	*Positive	Not tested	Not treated at time of study
5	50	Male	Equivocal	Not tested	Never treated
6	41	Male	Positive	Spotted fever group and typhus fever group	Began treatment after start of study
7	51	Female	*Positive	Not tested	Never treated
8	53	Female	Positive	Not tested	Previous treatment, but off at time of study
9	35	Female	Not tested	Not tested	Never treated
10	63	Female	Positive	Spotted fever group and Mycoplasma spp. (Labcorp)	Not treated at time of study
11	47	Female	*Positive	Not tested	Not treated at time of study
12	38	Male	Positive	None	On antibiotic therapy during study
13	43	Female	Positive	*Babesia* spp.	Treated but off antibiotics during study
14	53	Female	*Positive	Not tested	Never treated
15	48	Female	Clinical diagnosis, not tested	Not tested	Not treated at time of study
16	56	Female	Not tested	Not tested	Never treated
17	62	Female	*Positive	Not tested	Not treated at time of study
18	51	Female	Not tested	Not tested	Never treated
19	75	Female	**Positive	Not tested	Had 2 weeks treatment for Lyme but no treatment after
20	60	Female	**Positive	Not tested	Had 3 weeks erythromycin after EM rash and + serology, no treatment after
21	37	Female	Positive	Not tested	Not treated at time of study
22	53	Female	Not tested	Not tested	Not treated at time of study
23	68	Female	Not tested	Not tested	Never treated
24	73	Female	Not tested	Not tested	Never treated
25	58	Female	***Positive	Not tested	Never treated

All Lyme serology except that of patients 19, 20, and 25 tested and interpreted by IGeneX Reference Laboratories, Palo Alto, CA [10].

*Tested for *B. burgdorferi* after skin sampling was taken for study.

**Positive by 2-tiered CDC surveillance criteria.

***Tested by Spectracell Laboratories, Houston, TX.

A total of 25 patients were included in the study. Patients 1-5 were previously presented as case studies [6,7,9]. Clinical patient data is shown in Table 1. All patient samples were deidentified and coded, and all laboratory testing was performed in a blinded fashion.

### Cultures

Borrelia culture medium was prepared using Barbour–Stoner–Kelly H (BSK-H) complete medium with 6% rabbit serum (Sigma Aldrich, #B8291) and the following antibiotics: phosphomycin (0.02 mg/ml) (Sigma Aldrich), rifampicin (0.05 mg/ml) (Sigma Aldrich), and amphotericin B (2.5 μg/ml) (Sigma-Aldrich), as described previously [11]. Inocula for blood cultures were prepared as follows: Ten milliliters of whole blood were collected by venipuncture and left at room temperature for 10 to 15 minutes to allow clotting, followed by low speed centrifugation to separate red blood cells. Serum and some blood cells just below the serum layer were used as inocula. The serum/blood cell preparation was inoculated into the BSK medium.

For other cultures, whole scabs removed from the patient, skin scrapings from small lesions removed by a scalpel blade, or vaginal samples collected by swabbing inside the vagina with a sterile cotton-tipped swab were inoculated into the BSK medium. Cultures were incubated in an Oxoid anaerobic jar (Thermo Scientific) containing an AnaeroGen sachet (Thermo Scientific) to provide an anaerobic environment at 32°C. Culture fluid samples were examined by bright-field and/or dark-field microscopy for visible motile spirochetes weekly for up to 4 weeks. Cultures were processed for imaging and PCR by centrifuging the fluid at 15,000 g for 20 minutes to concentrate spirochetes, retaining the pellet and discarding the supernatant.

### Dieterle and anti-Bb immunostaining

Dermatological specimens and/or culture pellets from patients were processed for specialized staining at either McClain Laboratories LLC, Smithtown, NY; Interscope Laboratories, Interscope Pathology Medical Group, Canoga Park, CA; or the University of New Haven, West Haven, CT. Dieterle silver-nitrate staining was performed at either Interscope Laboratories or McClain Laboratories. Anti-Bb immunostaining was performed at McClain Laboratories or the University of New Haven.

For those samples submitted to McClain Laboratories for anti-Bb immunostaining, the following protocol was used: formalin-fixed, paraffin-embedded dermatological tissue and culture pellets were sectioned and immunostained using an unconjugated rabbit anti-Bb polyclonal antibody (Abcam ab20950) and then incubated with an alkaline phosphatase probe (Biocare Medical #UP536L) followed by a chromogen substrate (Biocare Medical #FR805CHC) and counterstained with hematoxylin. Positive and negative controls of both Dieterle and anti-Bb immunostains were prepared for comparison purposes using liver sections from uninfected mice and mice experimentally inoculated with Bb, as previously described [12]. Staining was titrated to define optimal antibody dilutions. For comparison purposes, controls of culture pellets from mixed Gram-positive bacteria and mixed Gram-negative bacteria, and sections of normal human skin were also examined to determine possible cross-reactivity with commonly encountered dermatological microorganisms.

For those samples submitted to the University of New Haven, anti-Bb immunofluorescent staining was performed as follows: formalin-fixed paraffin-embedded MD sections were processed for staining and imaging as previously described [13]. Dermatological specimens were formalin-fixed, blocked in paraffin and sectioned by McClain Laboratories. Culture fluid was fixed with acetone at -20°C onto SuperFrost™ slides (Thermo Fisher Scientific, Waltham, MA). Fixed specimens, both dermatological sections and fixed culture fluid, were pre-incubated with 10% normal goat serum (Thermo Fisher Scientific) in PBS containing 0.5% bovine serum albumin (BSA) (Sigma-Aldrich, St. Louis, MO) for 30 minutes to block non-specific binding of the secondary antibody. The slides were then washed with PBS containing 0.5% BSA and then incubated for 1 hour with fluorescein isothiocyanate (FITC)-labeled *Borrelia*-specific polyclonal antibody (Thermo Fisher Scientific, #73005) at a 1:50 dilution in PBS containing 1% BSA pH 7.4 followed by washing and then counterstained with 4’, 6-diamidino-2-phenylindole (DAPI) for 10 minutes. For the negative control samples, anti-specifically targeted antibody was replaced with normal rabbit IgG (Vector Laboratories, Burlingame, CA, #I–1000). Images were obtained using fluorescent microscopy.

### Electron microscopy

Samples for scanning electron microscopy (SEM) and transmission electron microscopy (TEM) were forwarded to the Electron Microscopy Facility, Department of Materials Science and Engineering, Clemson University, Anderson, South Carolina. Procedures were performed as previously described [6,7].

For SEM, culture pellets, fixed in buffered 2.5% glutaraldehyde were washed in buffer and dehydrated in a graded series of ethanol concentrations, followed by immersion in hexamethyldisilazane for 5–15 minutes and then air-dried at room temperature. Dry samples were mounted on Al mounts and were not coated but placed into a Hitachi TM3000 microscope (Tokyo, Japan) and imaged in the variable pressure mode.

For TEM, glutaraldehyde-fixed samples were washed in buffer and dehydrated in a graded series of ethanol concentrations. Samples were immersed in a 50:50 mixture of LR White embedding resin and 100% ethanol for 30 minutes, followed by pure LR White resin until they settled on the bottom of the vial. Resin-immersed samples were placed into pure resin in beam capsules and polymerized at 60°C overnight. Sections were cut on an Ultracut E microtome (Leica Microsystems, Wetzlar, Germany) producing sections 60–90 nm thick, and were placed onto copper grids then stained in uranyl acetate for 20 minutes. Imaging was performed using a Hitachi 7600 microscope.

### Molecular testing

#### PCR - University of New Haven

DNA was extracted from culture pellets and/or dermatological tissue by lysing overnight in 180 μl tissue lysis buffer (Qiagen) and 20 μl Proteinase K (Qiagen) at 56°C in a shaking water bath and phenol:chloroform extraction the following day. DNA was resuspended in 50-100 μl 1X TE buffer.

A published TaqMan assay targeting a 139-bp fragment of the gene encoding the *Borrelia* 16S rRNA was used for the detection of *Borrelia* in DNA extracted from patient samples [14]. Reactions were carried out in a final volume of 20 μl and consisted of 900 nM of each primer, 200 nM of probe, and 10 μl of 2X TaqMan Universal PCR Master Mix (Applied Biosystems). Amplifications were carried out on a CFX96 Real-Time System (Bio-Rad) and cycling conditions consisted of 50°C for 2 minutes, 95°C for 10 minutes, followed by 40 cycles of 95°C for 15 seconds and 60°C for 60 seconds. Fluorescent signals were recorded using CFX96 Real-Time software and Cq threshold was set automatically. Reactions were performed in triplicate. Positive and negative controls were run simultaneously. The positive control was *Bb sensu stricto* strain B-31. Four negative controls were used: water, normal human foreskin, normal skin from two Lyme patients, and normal skin from one Morgellons patient. *Borrelia* DNA was not detected in any of the negative controls.

Nested PCR primers for the 16S rRNA, flagellin (Fla), OspC, uvrA and pyrG genes were used as previously described [15-17]. Reactions were carried out in a final volume of 50 μl using 10 μl template DNA. Final concentrations were 2X Buffer B (Promega), 2 mM MgCl2, 0.4 mM dNTP mix, 2 μM of each primer, and 2.5 U Taq polymerase (Invitrogen). “Outer” primers were used in the first reaction. “Inner” primers were used for the nested reaction, in which 1 μl of PCR product from the first reaction was used as template for the second. Cycling parameters were as follows: 94°C for 5 minutes followed by 40 cycles of denaturation at 94°C for 1 minute, annealing for 1 minute (temperature based on the primer set used), and extension at 72°C for 1 minute, with a final extension step at 72°C for 5 minutes. PCR products were visualized on 1-2% agarose gels.

Sanger sequencing was used for gene analysis. PCR products were extracted from the agarose gels using the QIAquick Gel Extraction kit (Qiagen) according to the manufacturer’s instructions. The eluates from each sample were sequenced in both directions using the primers that generated the products. Obtained sequences were compared by searching the GenBank database (National Center for Biotechnology Information) using BLAST analysis. Sequence alignment (Clustel W) and neighbor-joining phylogenetic analyses were conducted using MEGA version 5. Tree support was evaluated by bootstrapping with 500 replications.

#### PCR - Australian biologics

Dermatological specimens and/or culture pellets were concentrated by centrifugation and stabilized with AL buffer (Qiagen). Samples were forwarded to Australian Biologics for *Borrelia* detection by real-time PCR targeting the 16S rRNA gene and endpoint PCR targeting the rpoC gene, as previously described [8,18]. The Eco™ Real-Time PCR system with software version 3.0.16.0 was used. DNA was extracted from the dermatological specimens and the culture pellets using the QIAamp DNA Mini Kit (Qiagen). Samples were analyzed in duplicate with positive and negative controls using primers for the *Borrelia* 16S rRNA and rpoC gene targets, as previously described [8,18]. Thermal profiles for all analyses were performed with incubation for 2 minutes at 50°C, polymerase activation for 10 minutes at 95°C then PCR cycling for 40 cycles of 10 secs at 95°C dropping to 60°C sustained for 45 secs. The magnitude of the PCR signal generated (∆R) for each sample was interpreted as either positive or negative as compared to positive and negative controls.

PCR products were visualized on 1-2% agarose gels and extracted from the gels using the QIAquick Gel Extraction kit (Qiagen) according to the manufacturer’s instructions. Sanger sequencing was used for gene analysis, as described previously [18].

#### Bb molecular beacons

Dr. Alan MacDonald designed the DNA sequences and generously donated the Bb molecular beacon DNA probes. Probe FlaB, a sequence of 23 nucleotides, was derived from the Bb open reading frame (ORF) BB0147 of the flagellin B gene that contains more than 1000 nucleotides. A nucleotide BLAST search of the 23 nucleotide probe sequence disclosed no matches other than that of Bb BB0147. Probe 740 was derived from the Bb ORF BB740 representing a Bb inner cell membrane protein, and a nucleotide BLAST search disclosed no matches other than that of the Bb ORF BB740.

Bb DNA staining and detection with molecular beacons was performed by the following protocol, as previously described [12]. Paraffin sections of dermatological specimens and culture pellets were completely dewaxed by baking at 60°C then immersed in serial 100% xylene baths, followed by serial immersion through 100% ethanol, 90% ethanol, 80% ethanol and distilled H_2_O, then air-dried. Fixed sections were immersed in 20 μl of working DNA beacon solution. Sectioned specimens were covered with plastic cut from a Ziploc® freezer bag then were heated at 90°C for 10 minutes to denature all DNA and RNA. Heat was reduced to 80°C for 10 minutes, then samples cooled gradually to room temperature. The stained slides were washed in PBS, and covered with 30% glycerol and a glass coverslip, then examined under an EPI Fluor microscope. Staining of research specimens was performed alongside staining of positive and negative controls. Positive controls consisted of a known Bb strain embedded in agarose, formalin-fixed and sectioned, as well as experimentally Bb-infected mouse liver sections. The negative control consisted of uninfected mouse liver sections.

## Results

### Histological examination – dermatological specimens

All patients were clinically diagnosed with MD by a healthcare provider based on the presence of skin lesions and/or skin crawling sensations with intradermal filaments that were visible with a hand-held microscope, as described in previous publications [4-9]. The dermatological material that met our diagnostic criterion was mainly in the form of calluses embedded with filaments, many of which were blue or red. MD calluses were easily removed from patients as they were composed of thickened skin that has separated from the dermis at the stratum basale. Histological examination of cross sections revealed epidermal layers from the stratum basale to the stratum corneum. In MD calluses, collagen and keratin filaments could be seen distributed throughout the epidermal tissue or projecting down from the stratum basale towards the dermis.

Dermatological specimens consisting of callus material from the following patients were submitted for histological sectioning and examination: 1-4, 8, 10-13, 15, 16, 18-20, 22, 23, 25. Biopsy sections were submitted for histological examination for patient 24. Sections of hair follicular bulbs and attached follicular sheaths rather than sectioned calluses were submitted for patient 5. Samples from patients 6, 7, 9, 14, 17 and 21 were used for culture and/or PCR detection only.

All histological sections were examined at 400X and 1000X magnification. In slides stained with Dieterle silver nitrate stain and/or anti-Bb antibodies, sectioned filaments embedded in epithelial cells were observed in samples of all callus material and in the biopsy material from patient 24. All filaments demonstrated the same morphology: a hollow medulla surrounded by a solid cortex. Filamentous material demonstrated no characteristics consistent with fungal elements such as hyphae, or any characteristics of known parasites such as microfilariae.

**Figure 2:**
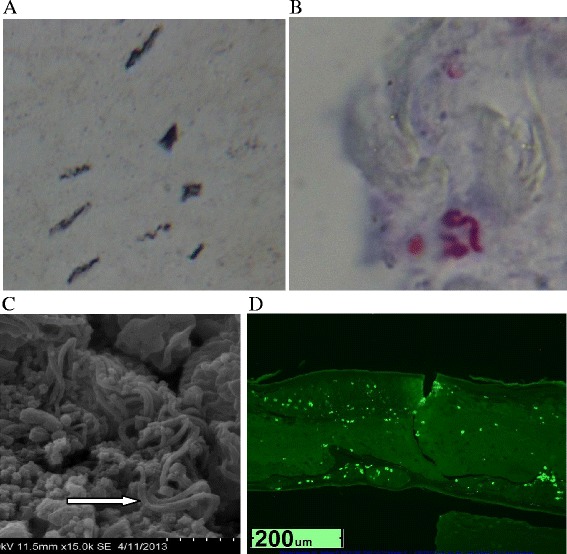
**Evaluation of skin samples from Morgellons disease patients. A**, Dieterle silver stain of skin sample from MD Patient 15 showing dark-staining spirochetes (1000x). **B**, Immunostaining of spirochete with anti-Bb antibody in skin sample from MD Patient 4 (1000x). **C**, Scanning electron micrograph of skin culture sample from MD Patient 6 showing wavy spirochetes (arrows). **D**, Hybridization of Bb-specific molecular beacon FlaB with spirochetes in skin sample from MD Patient 3 (400x).

Dieterle silver nitrate staining revealed bacteria morphologically consistent with *Borrelia* spirochetes in 17/19 patients (Table 2). Positively-stained spirochetes were observed in dermatological sections from the specimens submitted for patients 1-4, 8, 10-13, 15, 16, 18, 19, 20, 22 and 23 (Figure 2A). No spirochetes were observed for patients 5 or 24. Patient 5 had only sectioned follicular bulbs and sheaths submitted and patient 24 had biopsy sections submitted. These biopsy sections demonstrated bacterial forms consistent with cystic variants of Bb.

**Table 2:** **Microscopic examination of dermatological tissue sections with Dieterle or Warthin Starry silver nitrate stain and anti-Bb polyclonal immunostain**

**Patient #**	**Dieterle**	**Bb-polyclonal immunostain**	**Filaments morphologically consistent with MD**
1	Spirochetes observed	*Positive	Detected
2	Spirochetes observed	*Positive	Detected
3	Spirochetes observed	*Positive	Detected
4	Spirochetes observed	*Positive	Detected
5	Spirochetes not observed	**Positive	Detected
8	Spirochetes observed	Positive	Detected
10	Spirochetes observed	Positive	Detected
11	Spirochetes observed	Positive	Detected
12	Spirochetes observed	Positive	Detected
13	Spirochetes observed	Positive	Detected
15	Spirochetes observed	Positive	Detected
16	Spirochetes observed	Positive	Detected
18	Spirochetes observed	Positive	Detected
19	Spirochetes observed	Positive	Detected
20	Spirochetes observed	Positive	Detected
22	Spirochetes observed	Positive	Detected
23	Spirochetes observed	Positive	Detected
24	Spherules consistent with Bb morphological variants observed	Positive	Detected
25	Spirochetes observed	Positive	Detected

Dieterle and or Warthin-Starry silver nitrate staining performed at Interscope Laboratories, Los Angeles, CA, and or McClain Laboratories, Smithtown, NY.

Bb polyclonal immunostaining performed at McClain Laboratories.

*Polyclonal Bb immunostaining performed at University of New Haven, West Haven, CT and McClain Laboratories.

**Sectioned specimen consisted of hair follicular bulbs with attached root sheaths rather than skin. Polyclonal Bb immunostaining performed at the University of New Haven.

Anti-Bb immunohistochemical staining was reactive in all the histological sections of dermatological specimens submitted including the follicles and follicular sheaths from patient 5 and the two biopsy sections from patients 24 and 25 (100%). Individual spirochetes were discernible in many of these specimens (Figure 2B). Staining of Bb spirochetes was positive in infected mouse liver samples. There was no significant Bb immunostaining of the Bb-negative mouse liver control, the mixed Gram-positive bacterial pellet or normal human skin. Staining of sections of mixed Gram-negative bacteria from the Gram-negative culture pellet showed only weak background staining. In control studies of the anti-Bb immunostaining performed at the University of New Haven, the antibody reacted with Bb but not with *Treponema denticola*.

Bb spirochetes in a callus specimen from patient 2 were detected by SEM and on sectioned calluses from patients 1 and 2 by TEM as previously reported [6].

### Culture – general observations

Cultures were performed on skin, blood and/or vaginal specimens taken directly from patients 3, 4, 6-9, 13, 14, 17, 21 and 24. Motile viable organisms were observed in all cultures at 4 weeks of incubation except for the skin culture taken from patient number 6. Motile organisms displayed morphological variation, ranging from spherules to longer helical-shaped bacteria. None of the specimens could be subcultured onto blood agar, even in anaerobic conditions, and there was no contamination by commonly encountered aerobic Gram-positive bacteria. Some of the cultures had very few organisms, and documentation of growth by photography was difficult because of morphological variation. Some cultures were therefore concentrated by centrifugation and stained with Dieterle silver nitrate stain and anti-Bb immunostain for further characterization.

#### Skin culture

Dermatological specimens were submitted for culture from patients 3, 4, 6, 9, 13 and 21. Examination of culture fluid at 4 weeks incubation revealed motile spirochetes, except for the culture from patient 6. The culture from patient 9 revealed little growth, but one long motile spirochete was observed. Dieterle silver nitrate staining of skin cultures was performed on specimens from patients 3, 6, and 13. These specimens demonstrated positive staining of both spherules and spirochetes consistent with morphological forms of *Borrelia*. All specimens demonstrated strongly positive Bb polyclonal immunostaining of the bacteria present as well as the surrounding cellular debris. This may have been due to secreted exoantigens, antigens released by lysed Bb, or the presence of Bb intracellular infection. Data is summarized in Table 3.

**Table 3:** **Microscopic examination of sectioned culture pellets with Dieterle or Warthin Starry silver nitrate stain and anti-Bb polyclonal immunostain**

**Patient #**	**Inoculum type**	**Culture fluid**	**Dieterle**	Bb polyclonal immunostain*
3	Skin	Motile spirochetes	Spirochetes	Positive**
3	Blood	Motile bacteria	Spirochetes	Positive
4	Skin	Motile spirochetes	Not submitted	Not submitted
4	Blood	Motile bacteria	Not submitted	Not submitted
6	Skin***	Little growth observed	Spirochetes	Positive
7	Blood	Spherules	Spherules	Positive
8	Blood	Spherules	Spirochetes	Positive
8	Vaginal	Motile spherules	Spherules	Positive
9	Skin	Little growth, single long motile spirochete	Not submitted	Not submitted
13	Skin	Spherules/motile elongated bacteria	Spirochetes/ spherules	Positive
13	Blood	Spherules	Spirochetes/ spherules	Positive
14	Blood	Spherules	Not submitted	Not submitted
14	Vaginal	Motile spirochetes	Not submitted	Not submitted
17	Blood	Spherules	Spirochetes/ spherules	Positive
21	Skin	Spherules/ spirochetes	Not submitted	Not submitted
21	Vaginal	Motile spherules	Spherules	Positive
24	Blood	Spherules/ Motile bacteria	Not submitted	Not submitted
24	Vaginal	Motile spirochetes	Not submitted	Not submitted

*Anti-Bb immunostaining performed at McClain Laboratory, Smithtown, NY, except for the skin culture from patient 3.

**Anti-Bb immunostaining performed at the University of New Haven, West Haven, CT.

***SEM revealed well-defined spirochetes with periplasmic flagella.

The culture fluid from patient 6 did not reveal motile spirochetes, so to ascertain the presence of spirochetes a centrifuged culture pellet was submitted for SEM. SEM revealed that spirochetes with morphological features consistent with Bb were present in the culture pellet (Figure 2C).

#### Blood culture

Blood specimens for culture were taken from patients 3, 4, 7, 8, 13, 14, 17 and 24. Motile bacteria with spirochetal morphology were observed in culture fluid from the eight patients. Dieterle silver nitrate staining and anti-Bb immunostaining was performed on blood cultures concentrated by centrifugation from these patients. Patients 3, 7, 8, 13 and 17 demonstrated positive staining of both spirochetes and spherules consistent with morphological forms of *Borrelia* with Dieterle silver nitrate staining. These patients also demonstrated strongly positive Bb immunostaining of the bacteria in the samples as well as the surrounding cellular debris, as described above. Data is summarized in Table 3.

#### Vaginal culture

Swabs of vaginal secretions were submitted for culture from patients 8, 14, 21 and 24. Motile bacteria were visible in culture fluid from all four patients. Staining of vaginal cultures concentrated by centrifugation followed by Dieterle silver nitrate staining and anti-Bb immunostaining was performed for specimens taken from patients 8 and 21. Both specimens demonstrated positive staining of both spherules and spirochetes consistent with morphological forms of *Borrelia* with Dieterle silver nitrate stain. Both specimens demonstrated strongly positive Bb immunostaining of the bacteria in the samples as well as the surrounding cellular debris, as described above. Data is summarized in Table 3.

### Molecular testing

#### A. PCR Detection of *Borrelia*

Various sample types from 20 patients were submitted for PCR detection of *Borrelia* at three independent laboratories. These samples included whole dermatological calluses, histological skin sections, skin culture, blood culture, vaginal cultures, and one specimen of intestinal epithelial tissue that had sloughed off in the patient’s feces during an intestinal cleanse. *Borrelia* genes were detected in 18 of the patients whose samples were submitted, and results were equivocal for 2 patients. Amplicon sequences consistent with *Borrelia* DNA were obtained for the PCR products from 14 patients. *Bb sensu stricto* sequence was confirmed in 12 patients, while patient 23 was found to have an amplicon sequence consistent with *B. miyamotoi* and patient 24 had a sequence consistent with *B. garinii*. The latter patient had contracted Lyme disease in Europe. Positive PCR results are summarized in Table 4.

**Table 4:** **Detection of** ***Borrelia*** **DNA by PCR in samples derived from Morgellons patients**

**#**	**Specimen**	**PCR University of New Haven**	**PCR Australian Biologics**	**Sequencing**
1	Whole callus	pyrG, fla, ospC	16S rRNA	pyrG, fla, ospC
2	Whole callus	pyrG, fla, 16S rRNA	16S rRNA	pyrG
3	Whole callus	fla, pyrG, 16S rRNA, uvrA, ospC	16S rRNA	16S RNA, pyrG, fla, uvrA, ospC
3	Blood culture	fla		
4	Callus section	16S rRNA		
4	Blood culture	fla		
5	Whole callus	pyrG	16S rRNA	pyrG
6	Skin culture	16S rRNA		
7	Blood culture	fla		
8	Whole callus	pyrG, 16S rRNA, fla		pyrG, fla,
8	Blood culture	fla, pyrG		fla
9	Skin culture	fla		fla
10	Whole callus	16S rRNA, pyrG, fla	16S rRNA	16S rRNA, pyrG
10	Intestinal specimen		16S rRNA	
11	Whole callus	fla+/- indeterminate	Inhibited	
13	Whole callus	uvrA		uvrA
13	Blood culture	pyrG	16S rRNA	
13	Skin culture	pyrG		
14	Blood culture	fla	16S rRNA	fla
14	Vaginal culture	fla	16S rRNA	fla
15	Whole callus		16S rRNA	
16	Whole callus		rpoC	rpoC
17	Whole callus	pyrG, uvrA	16S rRNA	pyrG, uvrA
17	Blood culture	Real-time 16S rRNA +/- equivocal		
18	Whole callus	fla	16S rRNA	fla
21	Skin culture		16S rRNA +/- equivocal	
23	Whole callus		16S rRNA	rpoC*
24	Blood culture	fla	16S rRNA	
24	Vaginal culture		16S rRNA	rpoC**

*Sequence consistent with *B. miyamotoi.*

**Sequence consistent with *B. garinii.*

Amplicon sequences from all patients were consistent with *Bb sensu stricto* except for sequence from patient 23, which was consistent with *B. miyamotoi*, and patient 24, which was consistent with *B. garinii.*

Skin cultures from patients 6, 9, 13 and 21 were subjected to PCR testing, and three of the four samples tested positive, confirming the presence of *Borrelia* in the cultures. The fourth culture sample (patient 21) had equivocal PCR testing using the 16S rRNA probe but tested positive using the FlaB molecular probe (see below). Thus molecular testing confirmed the presence of viable *Borrelia* spirochetes in all four skin culture samples.

*Treponema denticola* was detected in 5/16 scab/callus samples sent to Australian Biologics. *T. denticola* was not detected in any blood, skin or vaginal cultures (data not shown).

#### B. In-situ DNA hybridization

Bb DNA was detected by staining with the fluorescent molecular probes FlaB and 740. Histological sections of callus material from patients 2, 3, 10, 11, 13, 15, 19, 20, 22 and sections of skin and/or vaginal culture pellets from patients 21 and 24 were stained with the FlaB probe. All these specimens were positively stained (Figure 2D). Histological sections of callus material from patients 1-4, 8, 10-13, 15, 16, and 18-20, and sections of the skin culture from patient 6 and blood culture from patient 7 were stained with probe 740. All of these specimens were positively stained. The results are summarized in Table 5.

**Table 5:** **Detection of Bb DNA by** ***in situ*** **hybridization with Bb-specific DNA probes in samples derived from Morgellons patients**

**Patient #**	**Specimen**	**Probe FlaB**	**Probe 740**
1	Callus	Not performed	Positive
2	Callus	Positive	Positive
3	Callus	Positive	Positive
4	Callus	Not performed	Positive
6	Skin culture	Not performed	Positive
7	Blood culture	Not performed	Positive
8	Callus	Not performed	Positive
10	Callus	Positive	Positive
11	Callus	Positive	Positive
12	Callus	Not performed	Positive
13	Callus	Positive	Positive
15	Callus	Positive	Positive
16	Callus	Not performed	Positive
18	Callus	Not performed	Positive
19	Callus	Positive	Positive
20	Callus	Positive	Positive
21	Skin culture	Positive	Not performed
21	Vaginal culture	Positive	Not performed
22	Callus	Positive	Not performed
24	Biopsy	Positive	Not performed

## Discussion

Despite compelling evidence to the contrary, MD continues to be attributed to delusions of parasitosis or delusional infestation [19-23]. The earlier studies demonstrating *Borrelia* spirochetes in MD dermatological specimens have involved only a small number of study subjects, and therefore a study involving a larger number of subjects was needed.

A major strength of our study is that MD patients were identified based on the presence of multicolored fibers within skin lesions or detectable under unbroken skin. Some of our patients did suffer from neuropsychiatric symptoms, and we do not deny that primary delusional infestation can occur in rare cases [1-4]. By selecting only MD patients meeting our dermopathy criterion, however, we have presumably excluded primary delusional infestation patients from our study. Although some MD patients suffering from neuropsychiatric symptoms with *Borrelia*-associated intradermal filaments may claim to have worms, parasites or the like, the skin crawling and stinging sensations that these patients feel coupled with visible skin lesions, anxiety and anthropomorphic thinking may result in complaints that are misinterpreted by clinicians as a primary delusional disorder. Other MD patients in our study had no neuropsychiatric symptoms and yet had the same *Borrelia*-associated dermopathy, so it appears that in our well-defined MD patient cohort these symptoms, when they occurred, were the result rather than the cause of the infectious dermopathy, as previously described [1-4,23].

We have provided evidence linking *Borrelia* infection with MD in a study group consisting of 25 patients. We detected *Borrelia* DNA by PCR and/or staining with Bb-specific DNA probes in 24/25 patients. We were able to demonstrate the presence of *Borrelia* in dermatological tissue and we were able to culture viable Bb spirochetes from skin, blood and vaginal secretions in some patients. The presence of spirochetes was confirmed by numerous testing methodologies, including culture, histology, anti-Bb immunostaining, electron microscopy, PCR and *in situ* Bb DNA hybridization. Laboratory testing methodologies were performed at five independent laboratories. Histological staining and electron microscopy of Morgellons dermatological tissue and cultures was performed at three different laboratories, while molecular testing for *Borrelia* was performed at three different laboratories using real-time PCR, nested PCR and *in situ* Bb DNA hybridization. Furthermore, *Borrelia* DNA amplicon sequencing was conducted at two different laboratories. We have thus provided corroborating evidence of *Borrelia* infection in MD patients that should be difficult to refute.

Recent studies reported similarities between MD and bovine digital dermatitis (BDD), a disease that is common in dairy herds [5,9]. BDD is characterized by skin lesions that commonly occur on and directly above the heel bulb of the hind feet of cattle [24,25]. Chronic BDD lesions demonstrate proliferative keratin filaments, and histological examination of diseased tissue reveals spirochetes identified as *Treponema* spp. dispersed among enlarged keratinocytes throughout the stratum spinosum and dermal papillae [26-30]. Though spirochetes are consistently detected in tissue from lesions, coinvolvement of other bacterial pathogens has been proposed as a contributing etiologic factor [27,28,31,32]. Treponemal spirochetes were confirmed as the primary etiologic agents when the condition was duplicated via experimental infection with pure cultured treponemes [33,34].

As with BDD, MD filaments are not textile fibers. MD fibers are biofilaments of human cellular origin produced by epithelial cells and stemming from deeper layers of the epidermis and the root sheath of hair follicles [5,9]. Immunohistochemical and histological staining has demonstrated that these multicolored filaments are composed of collagen and keratin [6,9]. They are nucleated at the base of attachment to adjacent epithelial cells, and the cells at the filament base are continuous in appearance with the surrounding skin cells [6]. Although the cause of coloration of red fibers has not been defined, the blue coloration is the result of melanin pigmentation rather than a dye, as shown by Fontana Masson histological staining [6]. There are no known textile fibers that are collagen in composition, nucleated at their base of attachment, or pigmented blue with melanin. Thus the characteristic fibers in MD are clearly distinct from textile fibers [6].

Histological sections of MD dermatological tissue reacted with anti-Bb immunostain in 19/19 of the dermatological specimens submitted for histological examination. Motile *Borrelia* spirochetes were cultured in medium inoculated with skin scrapings from 4 patients, thus demonstrating that *Borrelia* spirochetes in MD lesions are viable. *Borrelia* spirochetes were also detected in blood cultures from some MD patients in our study, confirming systemic Lyme borreliosis. Spirochetes characterized as strains of *Borrelia* were detected by PCR and/or in situ DNA hybridization in tissue or culture specimens from 24/25 patients; 15 of these patients had *Borrelia* gene products detected in dermatological specimens and/or skin cultures taken from MD lesions, and DNA amplicons from 14 patients were sequenced and confirmed to be *Borrelia* strains. Vaginal secretions from four patients were cultured, and three isolates were identified as *Borrelia* strains by PCR and in situ DNA hybridization.

Lyme borreliosis is a systemic infection that is commonly associated with dermatological manifestations [35]. Given that most MD patients are serologically reactive to Bb antigens, the presence of Lyme spirochetes in MD dermatological lesions is predictable and supports an etiologic role of the spirochetal disease. *Bb sensu stricto* and *Bb sensu lato* have been associated with numerous dermatological manifestations including erythema migrans, borrelial lymphocytoma, acrodermatitis chronica atrophicans, morphea, lichen sclerosus, cutaneous B-cell lymphoma, scleroderma, lymphadenosis cutis and prurigo pigmentosa [35-38]. Likewise it appears that MD is associated with Lyme disease in a subgroup of patients with this spirochetal illness [6-8]. It is possible that spirochetes other than *Bb sensu stricto* and the *Bb sensu lato* complex, such as the agent of syphilis, *Treponema pallidum,* could be responsible for similar manifestations in other patients. In support of this supposition, Ekbom’s original 1945 description of delusions of parasitosis reported that many of the patients in that study were diagnosed with syphilis, thus linking treponemal infection with pruritus, crawling sensations and belief of infestation [39]. Furthermore, treponemal spirochetes have been shown to induce the formation of filamentous lesions in animal models [26-30].

The mechanism of filament formation in MD is not yet elucidated. The filaments are composed of keratin and collagen and arise from proliferative keratinocytes and fibroblasts in human epithelial tissue [6,9]. Bb appears to have a predilection for fibroblasts and keratinocytes, and invasion of these cells by *Borrelia* spirochetes has been reported [40-42]. Bb appears to attach to fibroblasts followed by engulfment of the spirochetes, formation of vacuoles and intracellular replication [42]. Intracellular sequestration of Bb in skin fibroblasts and keratinocytes may protect the spirochetes from host defense mechanisms [40,41]. It is therefore reasonable to hypothesize that Bb intracellular infection of keratinocytes and fibroblasts may alter keratin and collagen expression and that the presence of *Borrelia* spirochetes in dermatological tissue is a primary etiologic factor in the evolution of MD lesions.

Viable Bb spirochetes have been isolated from lysates of fibroblast and keratinocyte monolayers treated with antibiotics [40,41]. Therefore, in addition to protection from host defenses, sequestration within fibroblasts and keratinocytes may protect Bb from antibiotic therapy. Spirochetes in MD dermatological tissue from Patients 1 and 12 were reactive to anti-Bb immunostains and we detected *Borrelia* DNA in dermatological tissue taken from these two patients. These patients were receiving aggressive antibiotic therapy at the time of this study. Patients 2, 8, 13, 19 and 20 had previously received antibiotic therapy for Lyme disease, yet still had detectable spirochetal infection. Persistent infection refractory to antibiotic treatment may therefore result from sequestration of *Borrelia* spirochetes within keratinocytes and fibroblasts in MD patients.

Although spirochetes appear to be the primary etiologic agents of MD, evidence suggests that the etiology is multifactorial. Secondary etiologic factors, such as female predominance, immune dysfunction, and other tickborne coinfections appear to play a role in the development of this dermopathy [1-5]. As noted in Table 1, we found serological evidence of tickborne coinfections including *Babesia, Anaplasma, Ehrlichia, Bartonella and Rickettsia* spp. in five of six patients who were tested. The role of these coinfections in MD remains undefined. Although we demonstrated the presence of *Borrelia* spirochetes in all of the patients in our study group, *T. denticola* was detected in dermatological specimens from five patients. The role of these commonly occurring oral spirochetes in the evolution of MD dermatological lesions and subsequent filament formation is uncertain, and we speculate that coinvolvement of these and perhaps other pathogens could be contributing or exacerbating factors in MD.

A study from the Centers for Disease Control and Prevention (CDC) concluded that pathogens were not involved in MD [22]. The search for spirochetal pathogens in that study was limited to Warthin-Starry staining on a small number of tissue samples and commercial two-tiered serological Lyme disease testing as interpreted by the CDC Lyme surveillance criteria [22]. It should be noted that only two of the patients in our study group were positive for Lyme disease based on the CDC Lyme surveillance criteria and yet *Borrelia* spirochetes were readily detectable in this group of 25 MD patients.

The diagnosis of Lyme disease is a controversial topic in the medical literature. Serological tests for Lyme disease lack sensitivity, and seronegativity has been demonstrated in patients with Bb infection [43,44]. PCR detection is not standardized, and sensitivity and specificity of testing therefore varies from laboratory to laboratory [45,46]. We detected *Borrelia* strains using different primers and different methodologies, and our findings show that primer hybridization differed between samples. Likewise *Borrelia* antigen detection may be unreliable, and immunostaining may lack sensitivity or specificity [46,47]. Although our dermatological and culture pellet sections were consistently reactive with anti-Bb polyclonal antibodies, we were not certain of the specificity of our testing. We wish to emphasize, however, that repeated detection of *Borrelia* spirochetes using a combination of diverse laboratory methods makes false-positive testing highly unlikely in these MD patients.

*Borrelia* culture is not available in many laboratories and can be challenging because of fastidious growth requirements and spirochetal pleomorphism [48,49]. The formation of spherical forms, truncated forms, straight forms, wavy forms and the like could result in positive cultures being overlooked. Our cultures demonstrated significant pleomorphism, and cystic or truncated variants were present, more so in blood cultures than skin cultures. In our experience histological identification is complicated because pleomorphism occurs in vivo as well as in vitro [50,51]. Sensitivity and specificity differences between laboratory methodologies to detect *Borrelia* spirochetes necessitate the use of several different methodologies to confirm the presence of *Borrelia* infection. If MD is determined to be pathognomonic for Lyme borreliosis it will aid in the diagnosis of Lyme disease in this group of patients. A recent study from Australia found MD in 6% of patients diagnosed with Lyme disease on that continent [52].

We achieved a high degree of success in detecting Bb and closely-related spirochetes from MD dermatological tissue. We attribute our success to several key factors. First, as stated previously we had a clear diagnostic criterion that allowed us to select the appropriate clinical cohort. Second, in contrast to *T. pallidum,* the treponemal agent of syphilis, *Borrelia* spp. can be cultured, thereby magnifying their numbers *in vitro* and increasing the opportunity for detection. Third, unlike secondary and tertiary syphilitic skin lesions where treponemes are seldom detected, we observed that MD lesions carry a high spirochetal load that allows for relatively easy detection, similar to lesions seen in cattle with BDD. Finally, we used a variety of sensitive microscopic and molecular methodologies to detect *Borrelia* spp., including molecular hybridization and PCR techniques that can detect spirochetal DNA in the picogram range.

The detection of *Borrelia* spirochetes in dermatological samples from a larger group of MD patients further validates the infectious nature of this dermopathy. As noted above, *T. pallidum* spirochetes are seldom detected in secondary and tertiary syphilitic skin lesions, even when sensitive molecular techniques such as PCR are performed, yet syphilis spirochetes are acknowledged to be the causative agent of these lesions [53-55]. The use of a clinical classification system for syphilis has helped with diagnosis and treatment of this systemic treponemal infection. In contrast to syphilis, we were able to consistently isolate and/or detect *Borrelia* spirochetes from MD dermatological specimens. Also in contrast to syphilis, no clinical classification system exists for MD.

Based on our experience with several hundred MD patients, we propose a clinical classification scheme that reflects the duration and location of MD lesions, as follows:

1. Early localized: lesions/fibers present for less than three (3) months and localized to one area of the body (head, trunk, extremities).
2. Early disseminated: lesions/fibers present for less than three (3) months and involving more than one area of the body (head, trunk, extremities).
3. Late localized: lesions/fibers present for more than six (6) months and localized to one area of the body (head, trunk, extremities).
4. Late disseminated: lesions/fibers present for more than six (6) months and involving more than one area of the body (head, trunk, extremities).

The classification scheme provides a medical framework that should help to validate and standardize the diagnosis of MD. Further studies are needed to determine whether this classification will have therapeutic and prognostic significance for MD patients.

## Conclusions

We undertook a detailed microscopic and molecular study of North American MD patients to investigate the presence of borrelial spirochetes systemically and in dermatological specimens. Based on culture, histology, immunohistochemistry, electron microscopy and molecular testing, we present extensive evidence for spirochetal infection in MD patients. Our study confirms that MD is a true somatic illness associated with Lyme disease. The proposed clinical classification scheme for MD should aid in the diagnosis and treatment of this complex illness.

## References

[1] Savely VR, Leitao MM, Stricker RB (2006). The mystery of Morgellons disease: Infection or delusion?. Am J Clin Dermatol.

[2] Savely G, Leitao MM (2005). Skin lesions and crawling sensation: disease or delusion?. Adv Nurse Pract.

[3] Savely VR, Stricker RB (2007). Morgellons disease: the mystery unfolds. Expert Rev Dermatol.

[4] Savely VR, Stricker RB (2010). Morgellons disease: analysis of a population with clinically confirmed microscopic subcutaneous fibers of unknown etiology. Clin Cosmet Investig Dermatol.

[5] Middelveen MJ, Stricker RB (2011). Filament formation associated with spirochetal infection: A comparative approach to Morgellons disease. Clin Cosmet Investig Dermatol.

[6] Middelveen MJ, Mayne PJ, Kahn DG, Stricker RB (2013). Characterization and evolution of dermal filaments from patients with Morgellons disease. Clin Cosmet Investig Dermatol.

[7] Middelveen MJ, Burugu D, Poruri A, Burke J, Mayne PJ, Sapi E (2013). Association of spirochetal infection with Morgellons disease. F1000 Res.

[8] Mayne P, English JS, Kilbane EJ, Burke JM, Middelveen MJ, Stricker RB (2013). Morgellons: a novel dermatological perspective as the multisystem infective disease borreliosis. F1000 Res.

[9] Middelveen MJ, Rasmussen EH, Kahn DG, Stricker RB (2012). Morgellons disease: A chemical and light microscopic study. J Clin Exp Dermatol Res.

[10] Shah JS, Du Cruz I, Narciso W, Lo W, Harris NS (2014). Improved sensitivity of Lyme disease Western blots prepared with a mixture of *Borrelia burgdorferi* strains 297 and B31. Chronic Dis Int.

[11] Bankhead T, Chaconas G (2007). The role of VlsE antigenic variation in the Lyme disease spirochete: persistence through a mechanism that differs from other pathogens. Mol Microbiol.

[12] Middelveen MJ, McClain SA, Bandoski C, Israel JR, Burke J, MacDonald AB (2014). Granulomatous hepatitis associated with chronic *Borrelia burgdorferi* infection: a case report. Research Open Access.

[13] Sapi E, Kaur N, Anyanwu S, Luecke DF, Datar A, Patel S (2011). Evaluation of in-vitro antibiotic susceptibility of different morphological forms of *Borrelia burgdorferi*. Infect Drug Resist.

[14] O'Rourke M, Traweger A, Lusa L, Stupica D, Maraspin V, Barrett PN (2013). Quantitative detection of *Borrelia burgdorferi* sensu lato in erythema migrans skin lesions using internally controlled duplex real time PCR. PLoS One.

[15] Margos G, Hojgaard A, Lane RS, Cornet M, Fingerle V, Rudenko N (2010). Multilocus sequence analysis of *Borrelia bissettii* strains from North America reveals a new Borrelia species. Borrelia kurtenbachii Ticks Tick Borne Dis.

[16] Sapi E, Pabbati N, Datar A, Davies EM, Rattelle A, Kuo BA (2013). Improved culture conditions for the growth and detection of Borrelia from human serum. Int J Med Sci.

[17] Clark KL, Leydet B, Hartman S (2013). Lyme borreliosis in human patients in Florida and Georgia. USA Int J Med Sci.

[18] Mayne PJ (2012). Investigations of *Borrelia burgdorferi* genotypes in Australia obtained from erythema migrans tissue. Clin Investig Dermatol Res.

[19] Lorenzo CR, Koo J (2004). Pimozide in dermatologic practice: A comprehensive review. Am J Clin Dermatol.

[20] Freudenmann RW, Lepping P (2009). Delusional infestation. Clin Microbiol Rev.

[21] Hylwa SA, Bury JE, Davis MD, Pittelkow M, Bostwick JM (2011). Delusional infestation, including delusions of parasitosis: Results of histologic examination of skin biopsy and patient-provided skin specimens. Arch Dermatol.

[22] Pearson ML, Selby JV, Katz KA, Cantrell V, Braden CR, Parise ME (2012). Clinical, epidemiologic, histopathologic and molecular features of an unexplained dermopathy. PLoS One.

[23] Stricker RB, Middelveen MJ (2012). Morgellons disease: More questions than answers. Psychosomatics.

[24] Cheli R, Mortellaro CM (1974). Digital dermatitis in cattle. *Proc 8th Int Meet Dis Cattle* Milan. Italy.

[25] Blowey RW, Sharp MW (1988). Digital dermatitis in dairy cattle. Vet Rec.

[26] Read DH, Walker RL, Castro AE, Sundberg JP, Thurmond JC (1992). An invasive spirochaete associated with interdigital papillomatosis of dairy cattle. Vet Rec.

[27] Borgmann JE, Bailey J, Clark EG (1996). Spirochete-associated bovine digital dermatitis. Can Vet J.

[28] Döpfer D, Koopmans A, Meijer FA, Szaskall I, Schukken WH, Klee W (1997). Histological and bacteriological evaluation of digital dermatitis in cattle, with special reference to spirochaetes and Campylobacter faecalis. Vet Rec.

[29] Read DH, Walker RL (1998). Papillomatous digital dermatitis (footwarts) in California dairy cattle: clinical and gross pathologic findings. J Vet Diagn Invest.

[30] Vink WD, Jones G, Johnson WO, Brown J, Demirkan I, Carter SD (2009). Diagnostic assessment without cut-offs: application of serology for the modeling of bovine digital dermatitis infection. Prev Vet Med.

[31] Grund S, Nattermann H, Horsch F (1995). Electron microscopic detection of spirochetes in digital dermatitis of cattle. Zentralbl Veterinarmed B.

[32] Demirkan I, Carter SD, Murray RD, Blowey RW, Woodward MJ (1998). The frequent detection of a treponeme in bovine digital dermatitis by immunohistochemistry and polymerase chain reaction. Vet Microbiol.

[33] Berry SL, Read DH, Famula TR, Mongini A, Döpfer D (2012). Long-term observations on the dynamics of bovine digital dermatitis lesions on a California dairy after topical treatment with lincomycin HCl. Vet J.

[34] Döpfer D, Anklam K, Mikheil D, Ladell P (2012). Growth curves and morphology of three Treponema subtypes isolated from digital dermatitis in cattle. Vet J.

[35] Malane MS, Grant-Kels JM, Feder HM, Luger SW (1991). Diagnosis of Lyme disease based on dermatologic manifestations. Ann Intern Med.

[36] Afa G, Caprilli F, Crescimbeni E, Morrone A, Progano G, Fazio M (1990). Anti-*Borrelia burgdorferi* antibodies in chronic erythema migrans, benign lymphadenosis cutis, scleroderma and scleratrophic lichen. G Ital Dermatol Venereol.

[37] Chao LL, Lu CF, Shih CM (2013). Molecular detection and genetic identification of *Borrelia garini* and *Borrelia afzelii* from patients presenting a rare skin manifestation of prurigo pigmentosa in Taiwan. Int J Infect Dis.

[38] Vasudevan B, Chatterjee M (2013). Lyme borreliosis and skin. Indian J Dermatol.

[39] Ekbom KA, Yorston G, Miesch M, Pleasance M, Rubbert S (2003). The pre-senile delusion of infestation. 1945. Translation: Hist Psychiatry.

[40] Georgilis K, Peacock M, Klempner MS (1992). Fibroblasts protect the Lyme disease spirochete, *Borrelia burgdorferi*, from ceftriaxone in vitro. J Infect Dis.

[41] Klempner MS, Rogers RA, Noring R (1993). Invasion of fibroblasts by the Lyme spirochete *Borrelia burgdorferi*. J Infect Dis.

[42] Chmielewski T, Tylewska-Wierzbanowska S (2010). Interactions between *Borrelia burgdorferi* and mouse fibroblasts. Pol J Microbiol.

[43] Stricker RB, Johnson L (2008). Serologic tests for Lyme disease: more smoke and mirrors. Clin Infect Dis.

[44] Dattwyler RJ, Volkman DJ, Luft BJ, Halperin JJ, Thomas J, Golightly MG (1988). Seronegative Lyme disease. N Engl J Med.

[45] Schmidt BL (1997). PCR in laboratory diagnosis of human *Borrelia burgdorferi* infections. Cin Microbiol Rev.

[46] Lange R, Seyyedi S (2002). Evidence of a Lyme borreliosis infection from the viewpoint of laboratory medicine. Int J Med Microbiol.

[47] Péter O, Bretz AG, Bee D (1995). Occurrence of different genospecies of *Borrelia burgdorferi* sensu lato in ixodid ticks of Valais. Switzerland Eur J Epidemiol.

[48] Mursic VP, Wanner G, Reinhardt S, Wilske B, Busch U, Marget W (1996). Formation and cultivation of *Borrelia burgdorferi* spheroplast-L-form variants. Infection.

[49] Brorson O, Brorson SH (2004). An in vitro study of the susceptibility of mobile and cystic forms of *Borrelia burgdorferi* to tinidazole. Int Microbiol.

[50] Embers ME, Barthold SW, Borda JT, Bowers L, Doyle L, Hodzic E (2012). Persistence of *Borrelia burgdorferi* in rhesus macaques following antibiotic treatment of disseminated infection. PLoS One.

[51] Meriläinen L, Herranen A, Schwarzbach A, Gilbert L. Morphological and biochemical features of *Borrelia burgdorferi* pleomorphic forms. *Microbiology.* published ahead of print January 6, 2015, doi:10.1099/mic.0.000027 10.1099/mic.0.000027 PMC4339653 25564498

[52] Mayne PJ (2015). Clinical determinants of Lyme borreliosis, babesiosis, bartonellosis, anaplasmosis, and ehrlichiosis in an Australian cohort. Int J Gen Med.

[53] Alessi E, Innocenti M, Ragusa G (1983). Secondary syphilis: Clinical morphology and histopathology. Am J Dermatopathol.

[54] Zoechiling N, Schluepen EM, Soyer HP, Kerl H, Volkenandt M (1997). Molecular detection of *Treponema pallidum* in secondary and tertiary syphilis. Br J Dermatol.

[55] Pereira TM, Fernandes JC, Viera AP, Basto AS (2007). Tertiary syphilis. Int J Dermatol.

